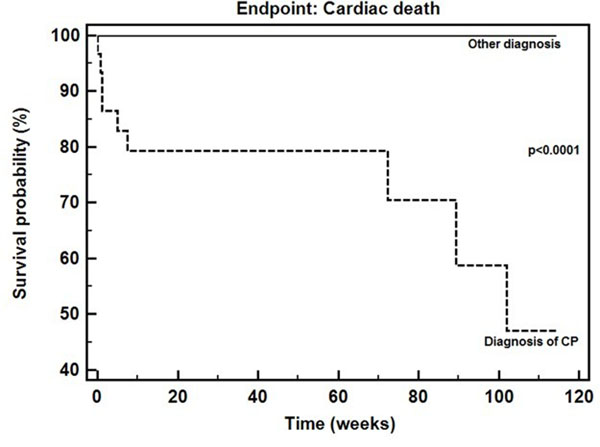# Cardiac magnetic resonance in pericardial disease

**DOI:** 10.1186/1532-429X-17-S1-P377

**Published:** 2015-02-03

**Authors:** Giovanni D Aquaro, Andrea Barison, Giancarlo Todiere, Alessandro Cagnolo, Michele Emdin, Massimo lombardi

**Affiliations:** 1 Fondazione Toscana G.Monasterio, Pisa, Italy; 2 Multimodality Cardiac Imaging Section, San Donato, Milano, Italy

## Background

Cardiac magnetic resonance (CMR) allows the detection of acute inflammation, fusion and thickening of pericardial layers and of effusion. We sought to evaluate the diagnostic and prognostic value of CMR in the setting of pericardial disease.

## Methods

CMR was performed in 146 patients (85 males, aged 56±16 years) with clinical suspicion of acute pericarditis (AP, n=46), pericardial effusion without inflammation (PE, n=47), and constrictive pericarditis (CP, n=53). Final diagnosis was ascertained by a combined evaluation of different CMR sequences. Patients were followed-up for all-cause and cardiac death.

## Results

The final diagnosis was AP in 56 patients (38%), PE in 38 (26%), and CP in 42 (29%). Pericardial disease was excluded in 7 patients (5%), with CMR evidence of abundant epicardial fat. The initial diagnosis was changed by CMR in 21% of patients. Initial suspicion was confirmed in 40/46(87%) patients with AP suspicion (alternative CP diagnosis: 2, PE: 1; no pericardial disease: 3). PE was confirmed by CMR in 35/47(75%) patients (10 with a final AP diagnosis, 2 patients with absent pericardial disease). A CP diagnosis was confirmed in 40/53(75%), while 6 presented signs of AP (resembling a "transient constrictive pericarditis"), 5 PE (as "effusive-constrictive pericarditis"), and 2 showed no pericardial disease. At Kaplan-Meyer analysis, on a 551-day median follow-up (IQ range 330-1110), patients diagnosed with CP had a worse prognosis than AP or PE patients (P <0.001).

## Conclusions

CMR has an additive value, as compared to conventional clinical evaluation, both for differential diagnosis of pericardial disease and for risk stratification.

## Funding

N/A.

**Figure 1:**